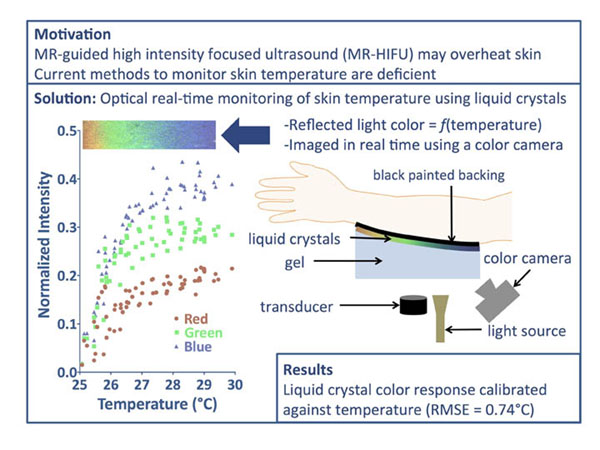# Optical measurement of skin temperature in MR-HIFU

**DOI:** 10.1186/2050-5736-3-S1-P81

**Published:** 2015-06-30

**Authors:** Daniel Yang, Haydar Celik, Doug Wackerle, David Kinnaird, Avinash Eranki, Matthew Oetgen, AeRang Kim, Karun Sharma, Harry Kim, Peter Kim, Pavel Yarmolenko

**Affiliations:** 1 Princeton University, Princeton, New Jersey, United States; 2 Children’s National Health System, Washington, D.C., United States; 3 The George Washington University School of Medicine, Washington, D.C., United States; 4 Texas Scottish Rite Hospital for Children, Dallas, Texas, United States

## Background/introduction

MR-guided high-intensity focused ultrasound (MR-HIFU) treatments may cause skin heating in the vicinity of the treatment site. Current MR thermometry methods do not provide reliable measurements of skin temperature either during the sonication or during the cool-down periods between sonications. These technical challenges require additional pauses to decrease the likelihood of skin burns, thus impacting treatment duration. Therefore, quantitative, accurate, and rapid techniques are needed to measure surface skin temperature during HIFU treatment. This study aims to develop an optical method that detects temperature changes at the skin surface to maintain a safe skin temperature during treatment and to reduce pauses between sonications.

## Methods

Chiral nematic liquid crystal slurry (LCR Hallcrest, LLC, Glenview, IL, #09-NSL33), which displays a change in reflected light color between 25-30°C was mixed with 0.4% w/v agarose gel at 80-85°C to create a 4 mL mixture of 10% liquid crystal slurry.

The mixture was poured onto a black-painted glass microscope slide and then covered with another glass slide (gel thickness = 0.5mm). Nickel-plated steel wire (220μm-thick) was used to heat the slide. Electrical current through the wire was adjusted to ensure a steady-state gradient across the glass slide that spanned from below 25°C to above 30°C. Color change of the liquid crystal gel layer was imaged using a DSLR camera and a stable light source placed at a 57° angle. A 0.2-mm diameter thermocouple (Omega Engineering, Inc., Stamford, CT, USA) sampled temperature at different distances from the heating wire to measure the temperature gradient. Local pixel color intensities around the thermocouple tip were isolated and spatially matched with measured temperatures using a custom MATLAB routine (Mathworks, Inc., Naticks, MA, USA). Multivariate polynomial regression was performed on the data to determine temperature as a function of red, green, and blue intensities (3rd order polynomials used for each).

## Results and conclusions

Color of the reflected light from the thermochromic liquid crystals varied with temperature in the agarose gel. As the temperature increased within the gel, red, green, and blue color intensities increase and level off at different rates. A multivariate polynomial regression showed RMS error of 0.74°C for the fit. Our data show that thermochromic liquid crystals provide an accurate, dynamic, and absolute method to monitor skin temperature during MR-HIFU treatment and ensure patient safety. While the liquid crystals used herein displayed a change of color in the 25-30°C range, crystals with other color-temperature ranges are commercially available and will be evaluated in further studies.

**Figure 1:**